# Deleterious localized stress fields: the effects of boundaries and stiffness tailoring in anisotropic laminated plates

**DOI:** 10.1098/rspa.2016.0391

**Published:** 2016-10

**Authors:** R. M. J. Groh, P. M. Weaver

**Affiliations:** Advanced Composites Centre for Innovation and Science, University of Bristol, Queen's Building, University Walk, Bristol BS8 1TR, UK

**Keywords:** boundary layer effects, composite laminates, higher-order plate theory, variable stiffness

## Abstract

The safe design of primary load-bearing structures requires accurate prediction of stresses, especially in the vicinity of geometric discontinuities where deleterious three-dimensional stress fields can be induced. Even for thin-walled structures significant through-thickness stresses arise at edges and boundaries, and this is especially precarious for laminates of advanced fibre-reinforced composites because through-thickness stresses are the predominant drivers in delamination failure. Here, we use a higher-order equivalent single-layer model derived from the Hellinger–Reissner mixed variational principle to examine boundary layer effects in laminated plates comprising constant-stiffness and variable-stiffness laminae and deforming statically in cylindrical bending. The results show that zigzag deformations, which arise due to layerwise differences in the transverse shear moduli, drive boundary layers towards clamped edges and are therefore critically important in quantifying localized stress gradients. The relative significance of the boundary layer scales with the degree of layerwise anisotropy and the thickness to characteristic length ratio. Finally, we demonstrate that the phenomenon of alternating positive and negative transverse shearing deformation through the thickness of composite laminates, previously only observed at clamped boundaries, can also occur at other locations as a result of smoothly varying the material properties over the in-plane dimensions of the laminate.

## 1. Introduction

In practical applications, composite laminates are typically modelled as thin plates and shells because the thickness dimension *t* is at least an order of magnitude smaller than representative in-plane dimensions *L*_*x*_ and *L*_*y*_. This feature allows the problem to be reduced from a three-dimensional (3D) to a two-dimensional (2D) one coincident with a chosen reference surface of the plate or shell, here defined in the *xy*-plane. The major advantage of this approximation is a significant reduction in the total number of variables and the associated computational effort. Such a theory is aptly called an equivalent single-layer theory (ESLT) because the through-thickness properties are condensed onto a reference layer by integrating properties of interest through the thickness, here denoted by the *z*-axis.

Many ESLTs are based on the axiomatic approach, whereby intuitive postulations of the displacement and/or stress fields in the thickness *z*-direction are made. Appropriate displacement-based, stress-based or mixed-variational formulations are then used to derive variationally consistent governing field equations and boundary conditions. Historically, the most popular axiomatic postulations use a pure displacement-based approach. These include the classical theory of plates developed by Kirchhoff [1] and then revisited by Love [2], and its extension to laminated structures, namely classical laminate analysis [3]. These theories assume that the effects of through-thickness deformations are negligible, in-plane displacement fields *u*_*x*_ and *u*_*y*_ vary linearly through the thickness, and the transverse displacement *u*_*z*_ is constant.

These classical theories are accurate for relatively thin structures (thickness to characteristic length ratios *t*/*L*≈1:100) but disregard higher-order distortions of the cross-section that occur for thicker structures and around local geometric features. Compared with isotropic structures, the analysis of layered composites is more cumbersome due to a plethora of additional factors. Transverse shear deformations are more pronounced in fibre-reinforced plastics because the ratio of longitudinal to shear modulus is approximately one order of magnitude greater (*E*_iso_/*G*_iso_=2.6, $E_{11}/G_{13}\approx \frac{140}{5}=28$, where 1 is in the fibre direction, 2 in the perpendicular resin direction and 3 in the through-thickness direction). The non-dimensional ratio *λ*=*E*/*G*(*t*/*L*)^2^ drives, what Everstine & Pipkin [4] called, the ‘stress channelling’ effect on in-plane stresses. As the non-dimensional ratio *λ* increases, the cross-section shears exceedingly and transitions from a constant rotation to a higher-order distortion field. Owing to the greater orthotropy ratios of *E*_11_/*G*_13_ and *E*_22_/*G*_23_ in composites, the ‘stress channelling’ effect is more pronounced in composite laminates than in isotropic plates. Finally, assuming perfect bonding of layers, interlaminar continuity of the displacements requires *u*_*x*_, *u*_*y*_ and *u*_*z*_ to be *C*^0^-continuous at interfaces but the interlaminar continuity of the transverse stresses forces the displacement fields to be *C*^1^-discontinuous [5]. This is known as the zigzag (ZZ) phenomenon due to the ‘zigzag’ shape of the displacements through the thickness.

Since the first half of the twentieth century, a number of models have been published that partially or completely revoke at least one of Kirchhoff's original assumptions. Examples include the first-order shear deformation theories of Mindlin [6], Yang *et al.* [7] and Shimpi [8], the third-order shear deformation theories of Levinson [9] and Reddy [10], and the shear and normal deformable theories of Hildebrand *et al.* [11] and Lo *et al.* [12]. However, these theories cannot explicitly capture ZZ effects as the in-plane variables *u*_*x*_ and *u*_*y*_ are defined to be at least *C*^1^-continuous in the *z*-direction.

In this regard, ESLTs that incorporate ZZ kinematics present a good compromise between local, layerwise accuracy and computational cost. Early ZZ theories were proposed in the Russian literature by Lekhnitskii [13] and Ambartsumyan [14]. Murakami [15] enhanced the first-order shear deformable theory by including a ZZ function that alternatively takes the values of +1 or −1 at layer interfaces. Motivated by the physical observation that layerwise differences in transverse shear moduli drive the ZZ effect, Tessler *et al.* [16] developed the refined ZZ theory (RZT). Here, the differences in transverse shear rigidities $G_{xz}^{\left(k\right)}$ and $G_{yz}^{\left(k\right)}$ of each layer *k*, and the average transverse shear rigidities *G*_*x*_ and *G*_*y*_ of the entire layup, define the layerwise ZZ slopes of the in-plane displacement fields *u*_*x*_ and *u*_*y*_, respectively. Accounting for ZZ deformations is critical for soft-core sandwich constructions but can also be significant for thick cross-ply [0°/90°] laminates [17].

A disadvantage of all previously mentioned displacement-based theories is that accurate recovery of transverse stresses from kinematic relations and constitutive equations is not guaranteed. For example, the derived transverse shear stresses typically violate the requirements of interfacial traction continuity. More accurate transverse stresses can be recovered *a posteriori* by integrating the in-plane stresses in Cauchy's 3D indefinite equilibrium equations [18]. The disadvantage of this technique is that the post-processed transverse stresses no longer satisfy the underlying governing field equations and are therefore variationally inconsistent.

This post-processing operation can be precluded by making some form of stress assumption. A particular class of model that we would like to highlight in this paper is based on the Hellinger–Reissner (HR) mixed variational principle. Here, the complementary form of the strain energy in terms of in-plane and transverse stresses is used, and Cauchy's 3D equilibrium equations are introduced as constraints via Lagrange multipliers. Reissner [19,20] was the first to use the HR principle to derive a new first-order theory for isotropic plates. As pointed out by Batra & Vidoli [21] and Batra *et al.* [22], one of the major advantages of using the HR principle is that independent assumptions of stress and displacement fields allow prescribed traction conditions to be satisfied exactly. In particular, this means that boundary layer effects and localized stress gradients towards boundaries can be captured accurately. In finite-dimensional displacement-based models, these conditions are approached asymptotically as the order of the model is refined.

By virtue of St. Venant's principle, classical analysis of laminates assumes purely planar stresses remote from geometric discontinuities. However, 3D stress concentrations that violate this principle do occur close to geometric features such as holes, notches, corners and free or constrained edges. For example, the presence of 3D stress fields in the vicinity of free edges is a well-established phenomenon. Early research into composite materials [23–25] showed that the elastic property mismatch between two adjacent laminae leads to a 3D stress concentration towards the free edge. The occurrence of this stress concentration occurs over a length approximately equal to the thickness of the laminate and is therefore a boundary layer effect. The comprehensive literature survey by Mittelstedt & Becker [26] collates a broad spectrum of approximate closed-form solutions based on elasticity theory as well as numerical methods, but also acknowledges that there is no universal method of dealing with the problem *a priori*.

Many studies [26] argue that a mathematical singularity in the interlaminar stresses occurs at the free edge. But because a singular stress state cannot actually exist in the real structure, the mathematical singularity must purely be an artefact of linear elasticity and the underlying assumptions of continuum mechanics, i.e. modelling the interlaminar interface via discrete changes in elastic properties. In reality, smooth transitions from layer to layer via a resin-dominated transition region must exist and plastic deformations within this region guarantee finite interlaminar stresses.

On the contrary, it has also been argued that the mathematical singularity occurs as a result of inappropriately modelling the traction-free boundary condition in a weak-form weighted-integral sense. Spilker [27] derived a special-purpose multilayered finite-element based on higher-order through-thickness expansions of stresses and displacements in each layer. In Spilker's element, equilibrium of stresses is guaranteed within each layer, and the equilibrium of tractions at layer interfaces and external surfaces (top and bottom surfaces and free edges) is satisfied exactly. Using this element the transverse shear and normal stresses exhibit steep gradients towards the free edge of a [0°/90°] laminate in tension but ultimately converge to a finite stress state, directly in conflict with the idea of a stress singularity at the free edge. Spilker [27] and Spilker & Chou [28] attributed this result to the exact point-by-point enforcement of stress equilibrium and free-edge traction conditions through the entire laminate thickness. When the free-edge condition was satisfied in a weighted-integral sense, i.e. the residual was forced to vanish on average over the free-edge cross-section, the singular stress field reappeared. Furthermore, the point-by-point stresses in the vicinity of the edge were inaccurate. Hence, Spilker & Chou [28] highlight the criticality of enforcing equilibrium and traction conditions exactly in order to accurately model boundary layers in the stress fields.

Compared with the free edge, the presence of boundary layers in the vicinity of supported edges has received less attention. Recently, Shah & Batra [29] investigated the stress distributions near edges of composite laminates using a displacement-based shear- and normal-deformable theory. A stress-recovery scheme for transverse stresses was used to compute accurate stress near the laminate edges, and the use of a weak-form finite-element solution implied singular stress fields at the laminate edges. The stress recovery scheme successfully predicted boundary layer effects in the transverse stress fields for moderately thick plates (*t*/*L*≥10) at 5% of the characteristic length from the edge compared with a 3D elasticity model. Most of the discrepancies occurred for ‘strong’ boundary conditions, such as clamped edges, and the authors did not investigate the influence of ZZ effects on the boundary solution.

Given the superior qualities of mixed formulations in predicting 3D stress fields, a higher-order theory derived from the HR principle is used herein to study boundary layers in laminated composites. The model is based on displacements and moments defined on an arbitrary reference plane and these variables facilitate an intuitive understanding of the underlying mechanics. The stress assumptions are inherently equilibrated and satisfy interlaminar and surface traction conditions, thereby following Spilker & Chou's [28] recommendation discussed above. The model is used to study boundary layer effects in the cylindrical bending of plates comprising constant stiffness laminae, e.g. traditional straight-fibre reinforced plastics, foam or honeycomb materials used in lightweight design, and also advanced variable-stiffness laminae which are manufactured by steering pre-impregnated fibre tows in curvilinear paths [30–33]. The relatively simple case of cylindrical bending is chosen here to clearly elucidate the governing drivers of the observed physical phenomena.

A second feature particular to the present work is that the governing field equations are solved in the strong form using the pseudo-spectral differential quadrature method. Differential quadrature is a numerical discretization technique proposed by Bellman *et al.* [34] that approximates the partial derivative of a field using a linear weighted sum of all the functional values in the domain, i.e. a differential analogue to integral quadrature. In this manner, any set of linear differential equations can be expressed as a linear system of algebraic equations by replacing the differential operators with the differential weighting matrix. The advantage of this strong-form method is that the underlying governing equations are satisfied exactly at all collocation points, including the boundary points, and not in a weighted-integral sense over the domain. Second, the use of a spectral Chebychev–Gauss–Lobatto grid biases the collocation points towards the boundaries and eliminates oscillations in the numerical solution that occur for equally spaced grids (the Runge effect). These particular features of the spectral mesh, coupled with strong-form solutions of the governing equations at each collocation point, allow accurate representations of the steep stress gradients towards clamped edges. For an in-depth survey of the differential quadrature method, we direct the reader to the recent review by Tornabene *et al.* [35].

The rest of the paper is structured as follows. Section 2 provides an overview of an HR formulation for the cylindrical bending of plates, which is a particular case of the generalized theory recently published by the present authors [36]. Section 3a investigates boundary layers in straight-fibre laminates towards clamped edges and characterizes the behaviour in terms of intuitive bending moment quantities. Section 3b considers stress fields in variable-stiffness laminates, and shows for the first time that non-intuitive localized stress fields that occur in the corners of constant-stiffness laminates can also occur in tow-steered laminates remote from any boundaries. Section 4 summarizes the present work and poses pertinent questions for future work.

## 2. Theory

**Figure 1.**
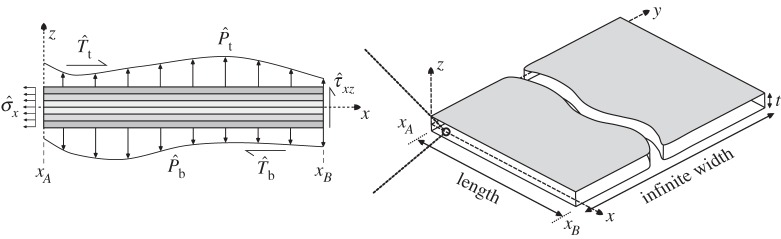
A wide composite plate loaded by distributed loads on the top and bottom surfaces and subjected to pertinent boundary conditions at ends *A* and *B*.

Consider a multilayered continuum, as represented in figure 1, undergoing static deformations under a specific set of externally applied loads and boundary conditions. The continuum has total thickness *t* and comprises *N*_*l*_ perfectly bonded laminae with layer thicknesses *t*^(*k*)^. The initial configuration of the plate is referenced in orthogonal Cartesian coordinates (*x*,*y*,*z*), with (*x*,*y*) defining the two in-plane dimensions and *z*∈[−*t*/2,*t*/2] defining the thickness coordinate. From hereon, we assume that the structural behaviour of this continuum is independent of the *y*-direction, as is the case in the cylindrical bending of an infinitely wide plate, such that a one-dimensional formulation can be used and the length of the plate corresponds to the dimension along the *x*-axis.

Within an equivalent single-layer framework, this multilayered structure is compressed onto a reference surface *Ω* coincident with the *xy*-plane by integrating the structural properties and 3D governing equations in the direction of the smallest dimension *z*. The plate undergoes static deformations under a specific set of externally applied shear and normal tractions $\left({\hat{T}}_{b},{\hat{P}}_{b}\right)$ and $\left({\hat{T}}_{t},{\hat{P}}_{t}\right)$ on the bottom and top surfaces of the 3D body, respectively, that are functions of the *x*-coordinate only.

### (a) Displacement field assumption

The equivalent single layer deforms according to the following generalized in-plane and transverse displacement field assumption,

2.1 $$\begin{array}{rl} u_{x}^{\left(k\right)}(x,z) & =u_{0}+z\theta +z^{2}\zeta +z^{3}\xi +\cdots +\phi^{\left(k\right)}(z)\psi \\ \;\text{and}\;u_{z}^{\left(k\right)}(x,z) & =w_{0} \end{array}\},$$

where *u*_0_ is the reference surface axial displacement, *θ* is the rotation of the plate cross-section, *ζ*,*ξ*,… are higher-order rotations, *ψ* is the ZZ rotation, and *ϕ*^(*k*)^ is a pertinent ZZ function of layer *k* which in this work is exclusively the RZT function defined by Tessler *et al.* [16]. In condensed matrix form, $u_{x}^{\left(k\right)}$ in equation (2.1) reads

2.2 $$u_{x}^{\left(k\right)}(x,z)=[{\text{f}}^{g}\;\phi^{\left(k\right)}]\left\{\begin{array}{c} U^{g}\\ \psi \end{array}\right\}={\text{f}}_{u}^{\left(k\right)}U,$$

where $U^{g}$ is a vector of global displacement fields and row vector ***f***^*g*^ describes the global through-thickness displacement variation. Hence,

2.3 $${\text{f}}^{g}(z)=[1\;z\;z^{2}\;z^{3}\;\ldots ],\;U^{g}={\left[u_{0}\;\theta \;\zeta \;\xi \;\ldots \right]}^{⊤},$$

where the superscript ⊤ denotes the matrix transpose. The in-plane strain is given by the first derivative of equation (2.2) in the *x*-direction. Thus, the linear axial strain component is

2.4 $$\epsilon_{x}^{\left(k\right)}=[{\text{f}}^{g}\;{\text{f}}^{l}]\left\{\begin{array}{c} \epsilon^{g}\\ \epsilon^{l} \end{array}\right\}={\text{f}}_{\epsilon }^{\left(k\right)}E,$$

where the global strain field *ϵ*^*g*^, local ZZ strain field *ϵ*^*l*^ and local ZZ row vector ***f***^*l*^ are given by

2.5 $$\epsilon^{g}=U_{,x}^{g},\;\epsilon^{l}={\left[\psi \;\psi_{,x}\right]}^{⊤},\;{\text{f}}^{l}(z)=[\phi_{,x}^{\left(k\right)}\;\phi^{\left(k\right)}]$$

and the comma notation denotes differentiation. Hence, the axial strain field in equation (2.4) is expressed as a combination of a global higher-order strain field (independent of local ply properties) and a local ZZ strain field (dependent on local ply properties).

### (b) Stress field assumption

The axial stress field is derived from the axial strain in equation (2.4) using the constitutive equation

2.6 $$\sigma_{x}^{\left(k\right)}={\bar{Q}}^{\left(k\right)}{\text{f}}_{\epsilon }^{\left(k\right)}E,$$

where ${\bar{Q}}^{\left(k\right)}$ is the reduced stiffness matrix, assuming a plane strain condition in the *y*-direction and a plane stress condition in the *z*-direction.

Next, the in-plane stress resultants are derived by integrating the axial stress of equation (2.6), weighted by the expansion functions ${\text{ f}}_{\epsilon }^{\left(k\right)}$, through the thickness of the plate

2.7 $$F=\int_{-t/2}^{t/2}{\text{f}}_{\epsilon }^{{\left(k\right)}^{⊤}}\sigma_{x}^{\left(k\right)}\;dz=\int_{-t/2}^{t/2}{\text{f}}_{\epsilon }^{{\left(k\right)}^{⊤}}{\bar{Q}}^{\left(k\right)}{\text{f}}_{\epsilon }^{\left(k\right)}\;dz\cdot E=\text{S}E,$$

where ***S*** is the higher-order laminate stiffness matrix of membrane and flexural rigidities with respect to the reference surface *Ω*. The first two terms of the stress resultant vector F are the classical membrane force *N* and bending moment *M*, and the following terms are higher-order moments.

The relation between stress resultants and strain variables equation (2.7) is now inverted to give

2.8 $$E=\text{s}F,\;\text{where}\;\text{s}={\text{S}}^{-1}\;\text{and}\;\text{S}=\int_{-t/2}^{t/2}{\text{f}}_{\epsilon }^{{\left(k\right)}^{⊤}}{\bar{Q}}^{\left(k\right)}{\text{f}}_{\epsilon }^{\left(k\right)}\;dz$$

and the in-plane stress of equation (2.6) recast in terms of stress resultants F

2.9 $$\sigma_{x}^{\left(k\right)}={\bar{Q}}^{\left(k\right)}{\text{f}}_{\epsilon }^{\left(k\right)}E={\bar{Q}}^{\left(k\right)}{\text{f}}_{\epsilon }^{\left(k\right)}\text{s}F.$$

Note, the advantage of expressing the in-plane stresses in terms of stress resultants rather than displacements is that the stresses are now functions of unknown variables rather than their derivatives, and this helps to reduce the order of the derived differential equations.

The transverse shear stress is derived by integrating the axial stress of equation (2.9) in Cauchy's axial equilibrium equation in the absence of body forces. This step yields

2.10 $$\tau_{xz}^{\left(k\right)}=-\int \frac{\partial \sigma_{x}}{\partial x}\;dz=\frac{\partial }{\partial x}[\{-{\bar{Q}}^{\left(k\right)}{\text{g}}^{\left(k\right)}+\alpha^{\left(k\right)}\}\text{s}F]+{\hat{T}}_{b},$$

where ***g***^(*k*)^ is the indefinite integral of ${\text{ f}}_{\epsilon }^{\left(k\right)}$ and captures the variation of $\tau_{xz}^{\left(k\right)}$ through the thickness of each ply *k*. Note, the derivative ∂/∂*x* applies to all terms within the square brackets in equation (2.10) as the material dependent quantities ${\bar{Q}}^{\left(k\right)}$, ***g***^(*k*)^ and ***s***, as well as the stress resultants F can vary over the dimensions of a variable-stiffness plate. The integration constants ***α***^(*k*)^ enforce the interfacial continuity conditions of the shear tractions and one of the prescribed surface conditions.

The transverse normal stress is derived in a similar fashion. Integrating the transverse shear stress of equation (2.10) in Cauchy's transverse equilibrium equation in the absence of body forces

2.11 $$\sigma_{z}^{\left(k\right)}=-\int \frac{\partial \tau_{xz}}{\partial x}\;dz=\frac{\partial^{2}}{\partial x^{2}}[\{{\bar{Q}}^{\left(k\right)}{\text{h}}^{\left(k\right)}-\alpha^{\left(k\right)}z+\beta^{\left(k\right)}\}\text{s}F]-{\hat{T}}_{b,x}(z-z_{0})+{\hat{P}}_{b},$$

where ***h***^(*k*)^ is the indefinite integral of ***g***^(*k*)^ and captures the variation of $\sigma_{z}^{\left(k\right)}$ through the thickness of each ply *k*. The integration constants ***β***^(*k*)^ enforce the interfacial continuity conditions of the normal tractions and one of the prescribed surface conditions. For explicit derivations of the integration constants ***α***^(*k*)^ and ***β***^(*k*)^, the interested reader is directed to [36,37].

Note, in the derivation of the integration constants of equations (2.10) and (2.11) the traction conditions are only enforced explicitly for one of the external surfaces. The condition on the other surface is automatically satisfied if the membrane and bending moment equilibrium equations

2.12 $$\begin{array}{rl} & N_{,x}+{\hat{T}}_{t}-{\hat{T}}_{b}=0\\ \;\text{and}\; & M_{,xx}+\frac{t}{2}({\hat{T}}_{t,x}+{\hat{T}}_{b,x})+{\hat{P}}_{t}-{\hat{P}}_{b}=0 \end{array}\}$$

are enforced when deriving the governing equations from the HR variational statement (see [17,37], for more details).

### (c) A contracted Hellinger–Reissner functional

The governing equations are derived by means of minimizing the potential energy functional *Π*_*HR*_ of the Hellinger–Reissner mixed-variational principle. For a 3D continuum independent of variations in the *y*-direction, the HR functional reads

2.13 $$\begin{array}{rl} \Pi_{\mathrm{HR}}(\text{u},\sigma ) & =\int_{V}U_{0}^{*}(\sigma_{ij})\;dV-\int_{S_{1}}{\hat{u}}_{i}t_{i}\;dS+\int_{V}u_{i}(\sigma_{ij,j}+f_{i})\;dV\\ & \;-\int_{S_{2}}u_{i}(t_{i}-{\hat{t}}_{i})\;dS,\;i,j=x,z, \end{array}$$

where, for linear elasticity with small strains and infinitesimal displacements, U*_0_(*σ*_*ij*_) is the complementary energy density expressed in terms of the Cauchy stress tensor *σ*_*ij*_. The displacements *u*_*i*_ are the Lagrange multipliers that enforce Cauchy's equilibrium equations *σ*_*ij*,*j*_+*f*_*i*_ throughout the volume of the continuum and also enforce the traction boundary conditions $t_{i}-{\hat{t}}_{i}$ on the boundary surface *S*_2_.

In this work, the model assumption of the axial displacements is as per equation (2.2), i.e. $u_{x}^{\left(k\right)}={\text{ f}}_{u}^{\left(k\right)}U$, whereas the transverse displacement *u*_*z*_=*w*_0_ is constant throughout the thickness. Thus, the term associated with Cauchy's equilibrium equations (ignoring body forces) in equation (2.13) is

2.14 $$\Pi_{L}=\int_{V}u_{i}\sigma_{ij,j}\;dV=\int_{V}\left[U^{⊤}{\text{f}}_{u}^{{\left(k\right)}^{⊤}}\left(\frac{\partial \sigma_{x}^{\left(k\right)}}{\partial x}+\frac{\partial \tau_{xz}^{\left(k\right)}}{\partial z}\right)+w_{0}\left(\frac{\partial \tau_{xz}^{\left(k\right)}}{\partial x}+\frac{\partial \sigma_{z}^{\left(k\right)}}{\partial z}\right)\right]dV.$$

Taking the first variation of this functional with respect to the displacement variables, i.e. δU and *δw*_0_, results in the higher-order equilibrium equations of the theory. By integrating the U-coefficient term in equation (2.14) by parts in the *z*-direction (note, U is independent of *z*), and then taking the first variation we have

2.15 $$\begin{array}{rl} \delta \Pi_{L_{1}} & =\iint_{-t/2}^{t/2}\delta U^{⊤}\left({\text{f}}_{u}^{{\left(k\right)}^{⊤}}\frac{\partial \sigma_{x}^{\left(k\right)}}{\partial x}-\frac{\partial {\text{f}}_{u}^{{\left(k\right)}^{⊤}}}{\partial z}\tau_{xz}^{\left(k\right)}\right)dz\;dx+\int \delta U^{⊤}{\text{f}}_{u}^{{\left(k\right)}^{⊤}}\tau_{xz}^{\left(k\right)}{|}_{-t/2}^{t/2}\;dx\\ & =\int \delta U^{⊤}\left[F_{,x}^{*}-T+{\text{f}}_{u}^{{\left(N_{l}\right)}^{⊤}}\left(z=\frac{t}{2}\right){\hat{T}}_{t}-{\text{f}}_{u}^{{\left(1\right)}^{⊤}}\left(z=\frac{-t}{2}\right){\hat{T}}_{b}\right]dx, \end{array}$$

where the vector of in-plane stress resultants $F^{*}$ and higher-order shear forces T is

2.16 $$F^{*}=\int_{-t/2}^{t/2}{\text{f}}_{u}^{{\left(k\right)}^{⊤}}\sigma_{x}^{\left(k\right)}\;dz,\;T=\int_{-t/2}^{t/2}\frac{\partial {\text{f}}_{u}^{{\left(k\right)}^{⊤}}}{\partial z}\tau_{xz}^{\left(k\right)}\;dz.$$

Setting the first variation to zero, the term in square brackets of equation (2.15) represents the set of equilibrium equations of the equivalent single-layer expressed in matrix form. These are the same higher-order equilibrium equations that result from the assumed displacement field if the principle of virtual displacements is applied. For a general assumption of displacements ***u*** and stresses ***σ***, the entire set of higher-order equilibrium equations in equation (2.15) needs to be satisfied. However, in this work the transverse shear stress and in-plane stress are inherently equilibrated, and this means that the equilibrium equations (2.15) are automatically satisfied *a priori* and do not need to be enforced in the HR variational statement as is shown below.

Integrating the transverse shear stress resultants T of equation (2.16) by parts we have

2.17 $$T={\text{f}}_{u}^{{\left(k\right)}^{⊤}}\tau_{xz}^{\left(k\right)}{|}_{-t/2}^{t/2}-\int_{-t/2}^{t/2}{\text{f}}_{u}^{{\left(k\right)}^{⊤}}\frac{\partial \tau_{xz}^{\left(k\right)}}{\partial z}\;dz.$$

As the transverse shear stress and axial stress balance explicitly in the present model, we can replace $\partial \tau_{xz}^{\left(k\right)}/\partial z$ with $-\partial \sigma_{x}^{\left(k\right)}/\partial x$ such that

2.18 $$T={\text{f}}_{u}^{{\left(N_{l}\right)}^{⊤}}\left(z=\frac{t}{2}\right){\hat{T}}_{t}-{\text{f}}_{u}^{{\left(1\right)}^{⊤}}\left(z=\frac{-t}{2}\right){\hat{T}}_{b}+\int_{-t/2}^{t/2}{\text{f}}_{u}^{{\left(k\right)}^{⊤}}\frac{\partial \sigma_{x}^{\left(k\right)}}{\partial x}\;dz.$$

Finally, by using the expression for $F^{*}$ in equation (2.16)

2.19 $$T={\text{f}}_{u}^{{\left(N_{l}\right)}^{⊤}}\left(z=\frac{t}{2}\right){\hat{T}}_{t}-{\text{f}}_{u}^{{\left(1\right)}^{⊤}}\left(z=\frac{-t}{2}\right){\hat{T}}_{b}+F_{,x}^{*}.$$

Thus, substituting equation (2.19) back into equation (2.15) the equilibrium equations in the square brackets vanish identically. The same arguments can be made for the second term in equation (2.14), namely $\partial \tau_{xz}^{\left(k\right)}/\partial x+\partial \sigma_{z}^{\left(k\right)}/\partial z$, but for brevity this is not elucidated in further detail herein.

However, the membrane and bending equilibrium equations (2.12) need to be satisfied to guarantee accurate recovery of the transverse stresses. To do this, these equilibrium equations (2.12) are enforced via two Lagrange multipliers, resulting in a *contracted* version of the HR principle.

Substituting all stress expressions equations (2.9)–(2.11) and their associated strains derived from the appropriate constitutive laws into the contracted functional gives

2.20 $$\begin{array}{rl} \Pi_{\mathrm{HR}} & =\frac{1}{2}\int_{V}U_{0}^{*}(F)\;dV-\int_{S_{1}}(\sigma_{x}{\hat{u}}_{x}^{\left(k\right)}+\tau_{xz}{\hat{w}}_{0})\;dS-\int_{S_{2}}\{u_{x}^{\left(k\right)}(\sigma_{x}-{\hat{\sigma }}_{x})+w_{0}(\tau_{xz}-{\hat{\tau }}_{xz})\}\;dS\\ & \;+\int u_{0}(N_{,x}+{\hat{T}}_{t}-{\hat{T}}_{b})\;dx+\int w_{0}\left(M_{,xx}+\frac{t}{2}({\hat{T}}_{t,x}+{\hat{T}}_{b,x})+{\hat{P}}_{t}-{\hat{P}}_{b}\right)dx, \end{array}$$

where ${\hat{u}}_{x}^{\left(k\right)}$ and ${\hat{w}}_{0}$ are the displacements defined on the boundary surface *S*_1_, and ${\hat{\sigma }}_{x}$ and ${\hat{\tau }}_{xz}$ are the tractions defined on the boundary surface *S*_2_.

Setting the first variation of equation (2.20) to zero, the resulting Euler–Lagrange field equations in terms of the functional unknowns *u*_0_, *w*_0_ and F are

2.21a $$\begin{array}{rl} & \delta u_{0}:\;N_{,x}+{\hat{T}}_{t}-{\hat{T}}_{b}=0 \end{array}$$

2.21b $$\begin{array}{rl} & \delta w_{0}:\;M_{,xx}+\frac{t}{2}({\hat{T}}_{t,x}+{\hat{T}}_{b,x})+{\hat{P}}_{t}-{\hat{P}}_{b}=0 \end{array}$$

2.21c $$\begin{array}{rl} \;\text{and}\; & \delta F^{⊤}:\;(\text{s}+\eta )F+\eta_{x}F_{,x}+\eta_{xx}F_{,xx}+\eta_{xxx}F_{,xxx}+\eta_{xxxx}F_{,xxxx}+{\hat{T}}_{b}\chi +{\hat{T}}_{b,x}\chi_{x}+{\hat{T}}_{b,xx}\chi_{xx}\\ & \;\;+{\hat{T}}_{b,xxx}\chi_{xxx}+{\hat{P}}_{b}\omega +{\hat{P}}_{b,x}\omega_{x}+{\hat{P}}_{b,xx}\omega_{xx}+\Lambda_{\mathrm{eq}}=0. \end{array}$$

The pertinent essential and natural boundary conditions are

2.22a $$\begin{array}{rl} \text{on }S_{1}\; & \delta F^{⊤}:\;\eta^{\mathrm{bc}}F+\eta_{x}^{\mathrm{bc}}F_{,x}+\eta_{xx}^{\mathrm{bc}}F_{,xx}+\eta_{xxx}^{\mathrm{bc}}F_{,xxx}+{\hat{T}}_{b}\chi^{\mathrm{bc}}+{\hat{T}}_{b,x}\chi_{x}^{\mathrm{bc}}+{\hat{T}}_{b,xx}\chi_{xx}^{\mathrm{bc}}+{\hat{P}}_{b}\omega^{\mathrm{bc}}\\ & \;+{\hat{P}}_{b,x}\omega_{x}^{\mathrm{bc}}+\Lambda_{{\mathrm{bc}}_{1}}={\hat{U}}_{\mathrm{bc}} \end{array}$$

2.22b $$\begin{array}{rl} \text{on }S_{1}\; & \delta F_{,x}^{⊤}:\;\rho^{\mathrm{bc}}F+\rho_{x}^{\mathrm{bc}}F_{,x}+\rho_{xx}^{\mathrm{bc}}F_{,xx}+{\hat{T}}_{b,x}\gamma_{x}^{\mathrm{bc}}+{\hat{P}}_{b}\mu^{\mathrm{bc}}+\Lambda_{{\mathrm{bc}}_{2}}=\hat{W}. \end{array}$$

2.22c $$\begin{array}{rl} \;\text{and}\;\text{on }S_{2}\; & \delta U^{⊤}:\;F^{*}={\hat{F}}^{*}\;\text{and}\;\delta w_{0}:\;Q=\hat{Q}, \end{array}$$

where *Q* is the classical transverse shear force. Note, the full derivation of the above governing equations, including details of all coefficients is available in references [37,38].

The governing field equations and boundary conditions related to $\delta F^{⊤}$ are expressed in vectorial notation, with each row defining a separate equation. Equation (2.21c) is an enhanced version of the classical constitutive equation

2.23 $$\left\{\begin{array}{c} u_{0,x}\\ -w_{0,xx} \end{array}\right\}={\left[\begin{array}{cc} A & B\\ B & D \end{array}\right]}^{-1}\left\{\begin{array}{c} N\\ M \end{array}\right\}=\text{s}F$$

with *u*_0,*x*_ and −*w*_0,*xx*_ the reference surface stretching strain and curvature, respectively, but additionally accounting for higher-order effects in ***s*** multiplied by derivatives of F, akin to the stress-gradient approach of non-local elasticity. The members of ***η*** are correction factors related to transverse shear stresses and transverse normal stresses. Similarly, members of row vectors ***χ*** and ***ω*** are correction factors related to the surface shear and normal tractions, respectively. The terms ***ρ***, ***γ*** and ***μ*** are transverse normal correction factors on the boundary.

The column vectors ***Λ*** only include the Lagrange multipliers *u*_0_, *w*_0_ and their derivatives. Specifically,

2.24a $$\begin{array}{rl} \Lambda_{\mathrm{eq}} & ={\left[-u_{0,x}\;w_{0,xx}\;0\;\ldots \right]}^{⊤} \end{array}$$

2.24b $$\begin{array}{rl} \Lambda_{\mathrm{bc}1} & ={\left[u_{0}\;-w_{0,x}\;0\;\ldots \right]}^{⊤}\;\text{and}\;{\hat{U}}_{\mathrm{bc}}={\left[{\hat{U}}^{g}\;0\;\hat{\psi }\right]}^{⊤} \end{array}$$

2.24c $$\begin{array}{rl} \;\text{and}\;\Lambda_{\mathrm{bc}2} & ={\left[0\;w_{0}\;0\;\ldots \right]}^{⊤}\;\text{and}\;\hat{W}={\left[0\;{\hat{w}}_{0}\;0\;\ldots \right]}^{⊤}. \end{array}$$

## 3. Results and discussion

A third-order ZZ version of the model derived in §2 denoted by HR3-RZT (expansion up to *z*^3^ and using the RZT ZZ function [16]) is used from hereon to study boundary layer effects at clamped edges of constant-stiffness laminates. Furthermore, the model is used to investigate localized stress fields driven by variations in material stiffness along the plate length.

### (a) Boundary layers towards clamped edges

**Figure 2.**
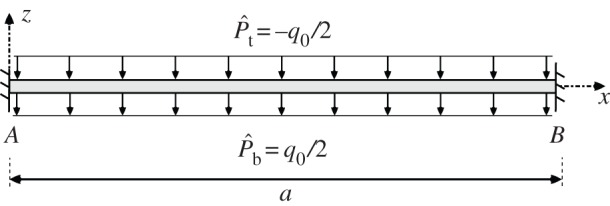
A multilayered wide plate clamped at both ends and loaded by a uniform pressure on the top and bottom surfaces. The total pressure acting on the plate is ${\hat{P}}_{t}-{\hat{P}}_{b}=-q_{0}$.

We consider a wide multilayered plate clamped at two ends *x*_*A*_=0 and *x*_*B*_=*a* with a pressure load equally divided between the top and bottom surfaces, i.e. ${\hat{P}}_{b}=-{\hat{P}}_{t}=q_{0}/2$ as shown in figure 2, with a plane strain condition in the lateral *y*-direction. As shown in equation (2.21b), the net pressure is given by ${\hat{P}}_{t}-{\hat{P}}_{b}$ and hence the total pressure is equal to −*q*_0_.

The HR3-RZT model has two displacement unknowns *u*_0_ and *w*_0_, and five stress unknowns $F={\left(N,M,O,P,L\right)}^{⊤}$, where *N* and *O* are the classic and second-order membrane stress resultants, respectively; *M* and *P* are the classic bending moment and second-order bending moment, respectively; and *L* is the ZZ moment.

We consider two laminates with stacking sequences shown in table 1, where laminate 1 is a non-symmetric composite laminate, and laminate 2 is a non-symmetric sandwich panel. Both plates have thickness-to-length ratio *t*/*a*=1:10 and comprise materials p and pvc defined in table 2. Material p represents an orthotropic material that was introduced by Pagano [39] which has since been used by various researchers in model validation studies. Material pvc represents an isotropic foam that is used in the sandwich panel.

**Table 1.** Composite laminate and sandwich plate stacking sequences with *t*/*a*=1:10 used to investigate boundary layers. Subscripts indicate the repetition of a property over the corresponding number of layers.

laminate	*t*/*a*	thickness ratio	material	stacking sequence
1	1:10	[(1/4)_4_]	[p_4_]	[0/90/0/90]
2	1:10	[(1/8)_2_/0.5/(1/8)_2_]	[p_2_/pvc/p_2_]	[0/90/0_2_/90]

**Table 2.** Mechanical properties of materials p, pvc and IM7. Material p is commonly used in the 3D elasticity solutions of Pagano [39].

material	*E*_1_	*E*_2_	*E*_3_	*G*_12_	*G*_13_	*G*_23_
p	25×10^6^	1×10^6^	1×10^6^	5×10^5^	5×10^5^	2×10^5^
pvc	25×10^4^	25×10^4^	25×10^4^	9.62×10^4^	9.62×10^4^	9.62×10^4^
IM7	163×10^9^	12×10^9^	12×10^9^	5×10^9^	4×10^9^	3.2×10^9^
material	*ν*_12_		*ν*_13_		*ν*_23_	
p	0.25		0.25		0.25	
pvc	0.3		0.3		0.3	
IM7	0.3		0.3		0.3	

Using the pseudo-spectral differential quadrature method (see §1) the governing differential equations are converted into algebraic ones by replacing the differential operators with weighting matrices that operate on all functional unknowns within the domain. In this work, the numerical solution procedure was implemented in Matlab. The number of collocation points in the discretization grid was increased until the absolute maximum value of all stress fields at one end of the plate converged to within 0.5%. As the grid points are biased towards the boundaries in the Chebychev–Gauss–Lobatto grid and due to the fact that stresses are treated as unknowns in the present HR formulation, convergence occurs relatively quickly. All results that follow are based on a grid of 45 collocation points.

The HR3-RZT model is validated numerically using a 3D finite-element method (FEM) model. The plate was modelled in the commercial software package Abaqus using a 3D body 1000 mm long, 100 mm thick and 1000 mm wide that was meshed with 96 000 C3D8R brick elements, i.e. 160 elements through the thickness, 600 elements along the length and one element across the width. The plane strain condition in the width direction was enforced by using a single element in this direction and by restraining the lateral edges from expanding. A load of ${\hat{P}}_{b}=-{\hat{P}}_{t}=50\;\mathrm{kPa}$ was applied as a pressure loading on the top and bottom surfaces. Finally, all nodal degrees of freedom were constrained throughout the thickness of the two clamped edges.

The three stress fields *σ*_*x*_, *τ*_*xz*_ and *σ*_*z*_ are normalized using the expressions

3.1 $$\bar{w}=\frac{10^{6}t^{2}}{q_{0}a^{4}}\int_{-t/2}^{t/2}u_{z}\left(\frac{a}{2},z\right)dz,\;{\bar{\sigma }}_{x}=\frac{t^{2}}{q_{0}a^{2}}\sigma_{x}\left(\frac{a}{2},z\right),\;{\bar{\tau }}_{xz}=\frac{1}{q_{0}}\tau_{xz}(0,z),\;{\bar{\sigma }}_{z}=\frac{1}{q_{0}}\sigma_{z}\left(\frac{a}{2},z\right).$$

**Figure 3.**
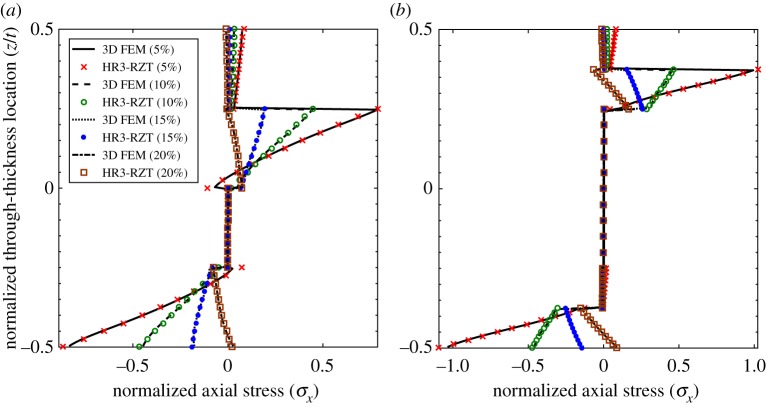
Through-thickness plot of normalized axial stress ${\bar{\sigma }}_{x}$ at locations 5%, 10%, 15% and 20% of the plate span from clamped end *x*_*A*_. (*a*) Laminate 1 and (*b*) laminate 2. (Online version in colour.)

**Figure 4.**
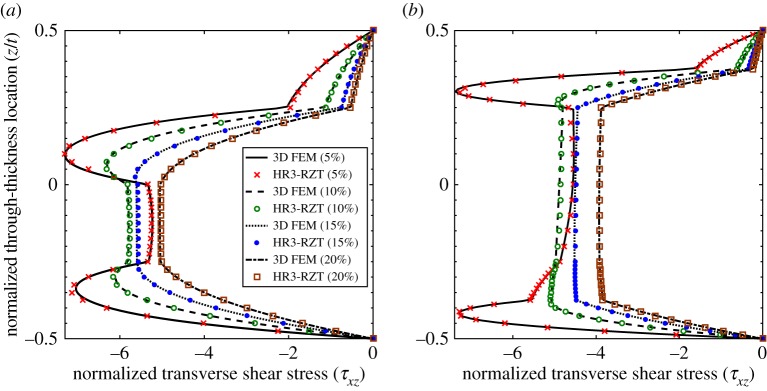
Through-thickness plot of normalized transverse shear stress ${\bar{\tau }}_{xz}$ at locations 5%, 10%, 15% and 20% of the plate span from clamped end *x*_*A*_. (*a*) Laminate 1 and (*b*) laminate 2. (Online version in colour.)

**Figure 5.**
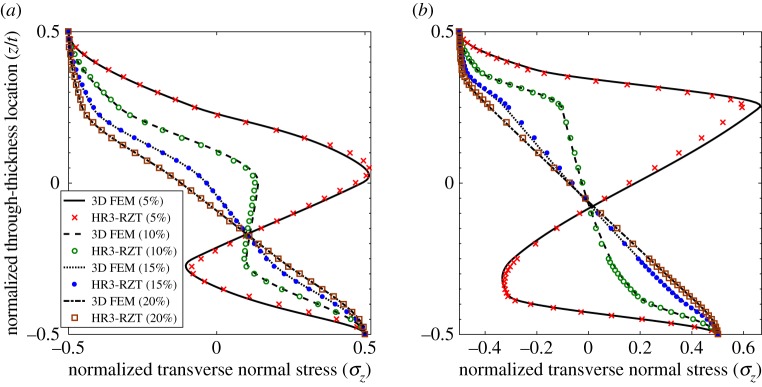
Through-thickness plot of normalized transverse normal stress ${\bar{\sigma }}_{z}$ at locations 5%, 10%, 15% and 20% of the plate span from clamped end *x*_*A*_. (*a*) Laminate 1 and (*b*) laminate 2. (Online version in colour.)

The through-thickness distributions of the three stress fields are plotted in figures 3–5 at four locations 5%, 10%, 15% and 20% of the plate length from the clamped end *x*_*A*_. The nearest position of 5% to the clamped end was chosen to minimize the effect of the boundary on the displacement-based 3D FEM stress results which are not guaranteed to converge at the edge. The stress plots of HR3-RZT and 3D FEM correlate well in figures 3–5.

Consider the results for laminate 1. Figures 3*a*, 4*a* and 5*a* clearly show the changes in the through-thickness profiles of the stress fields at different distances from the clamped edge. The through-thickness profile of ${\bar{\tau }}_{xz}$ at the 20% location (figure 4*a*) represents the converged solution free of boundary layer effects. Close to the clamped ends, e.g. at the 5% location, the clamped condition induces a boundary layer and this causes a change in shape of the transverse shear stress profile.

Between the 20% and the 5% curves in figure 4*a*, the maximum magnitude of ${\bar{\tau }}_{xz}$ redistributes from the midplane towards the surfaces causing a reversal in the transverse shear stress profile with smaller stress magnitudes towards the midplane. The same behaviour was previously observed for sandwich beams (see figs 11b and 12b in [17]), and occurs because of the ZZ effect. For the sandwich beams in [17], the extremely soft internal core cannot support the transverse shear stress magnitude in the stiff outer layers and this causes a redistribution of the loads from the core to the outside surfaces. Generally speaking, ZZ effects do not affect non-sandwich laminates, such as the [0/90/0/90] stacking sequence investigated here, to the same extent as sandwich laminates. Therefore, an analyst may argue that ZZ deformations can be ignored for non-sandwich laminates to improve computational efficiency. However, as observed for laminate 1 in figure 4*a*, this is not so as the clamped support condition exacerbates the ZZ deformations towards the boundaries.

The same effect also occurs in the transverse normal stress plots of figure 5*a*. The transverse normal stress field at the 20% location represents the converged solution free of boundary layer effects. Closer to the clamped boundary at the 5% location the through-thickness variation of ${\bar{\sigma }}_{z}$ changes considerably due to increased ZZ deformations.

The plots of axial stress in figure 3*a* show that ‘stress channelling’ decreases towards the boundary. This manifests itself by a reduction of the cubic *z*-wise variation of ${\bar{\sigma }}_{x}$ between the 20% and the 5% location. This is because at the 5% location the additional linear behaviour of the ZZ effect reduces the relative magnitude of the cubic through-thickness variation.

Similarly, the plots for sandwich laminate 2 (figures 3*b*, 4*b* and 5*b*) show similar trends for the through-thickness distributions of the three stress fields. Towards the boundaries the increasing effect of ZZ deformations causes transverse shear loads to be redistributed from the plate midplane to the surfaces. In fact, the redistribution of the transverse stresses can be explained intuitively using Cauchy's equilibrium equations. Given that the clamped support causes localized axial stress gradients in *σ*_*x*_, this rate of change of ∂*σ*_*x*_/∂*x* must be balanced by a rate of change ∂*τ*_*xz*_/∂*z*. Hence, the stress gradients of the axial stress alter the through-thickness profile of the transverse shear stress. In the same manner, the rate of change of *τ*_*xz*_ in the *x*-direction leads to an increase in the *z*-wise rate of change of *σ*_*z*_.

**Figure 6.**
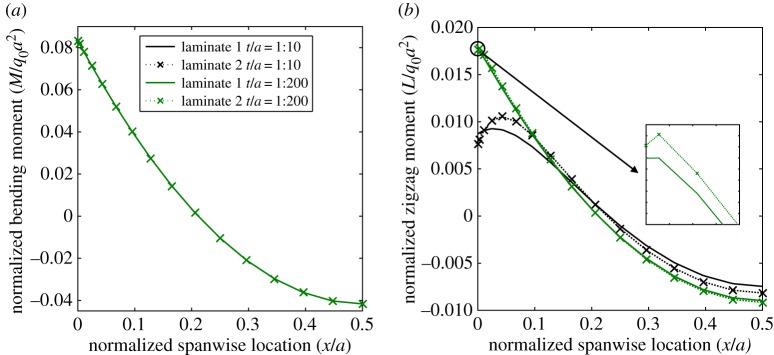
Normalized bending moment *M*/*q*_0_*a*^2^ and ZZ moment *L*/*q*_*o*_*a*^2^ for two different laminates plotted against normalized axial location *x*/*a* for different thickness-to-length ratios *t*/*a*. Only half of the plot is shown due to the symmetry condition at *x*/*a*=0.5. The insert shows the boundary layers of the thinner plate for *x*/*a*∈[0,0.02]. (*a*) Bending moment, *M*, and (*b*) ZZ moment, *L*. (Online version in colour.)

The extent of the boundary layers along the lengths of laminates 1 and 2 are shown graphically in figure 6. The plots show the variation of normalized bending moment *M*/(*q*_0_*a*^2^) (figure 6*a*) and ZZ moment *L*/(*q*_0_*a*^2^) (figure 6*b*) along the length of the plates. The plots only show half of the plate span *x*/*a*∈[0,0.5] due to the symmetry condition at *x*/*a*=0.5.

According to the equilibrium of moments and transverse forces, the bending moments *M* for laminates 1 and 2 are prescribed fully by the loading and boundary conditions and are independent of stacking sequence. Thus, the bending moments show no observable local variations towards the boundaries. However, the distributions of ZZ moment *L* are not the same due to the different degrees of transverse anisotropy inherent in the stacking sequences. Furthermore, we observe a boundary layer effect as *L* transitions from the global sinusoidal curve remote from the edge to a local high-order behaviour towards the edge.

The boundary layer effects observed for laminates 1 and 2 in figures 3–5 are higher-order phenomena that depend on the rate of change of the ZZ deformations within the structure. The boundary layer scales with the thickness-to-length ratio *t*/*a* and the degree of transverse shear modulus anisotropy. For example, consider laminates 1 and 2 with reduced thickness-to-length ratio *t*/*a*=1:200. The axial plots of the ZZ moment *L* for these two thinner configurations are shown alongside the plots for the originally thicker configurations (*t*/*a*=1:10) in figure 6*b*. For both cases, the overall magnitude of the ZZ moment *L* (figure 6*b*) is comparable to the classical bending moment *M* (figure 6*a*) and thus the ZZ moment plays a crucial role in the static behaviour of the plate.

However, the boundary layer itself is not driven by the overall magnitude of the ZZ moment but rather by localized changes with respect to the classic sinusoidal curve that governs the behaviour in the absence of boundary layer effects. For the thinner configuration, the localized change in the ZZ moment curve, characterized by the presence of an inflection point, occurs significantly closer to the edge. Furthermore, the curvature of the ZZ moment curve, and thereby the effect of the boundary layer on the stress profiles, also reduces for the thinner configuration. Hence, reducing the thickness-to-length ratio has not affected the overall magnitude of the ZZ moment close to the edge but eliminated the boundary layer effects associated with it.

**Figure 7.**
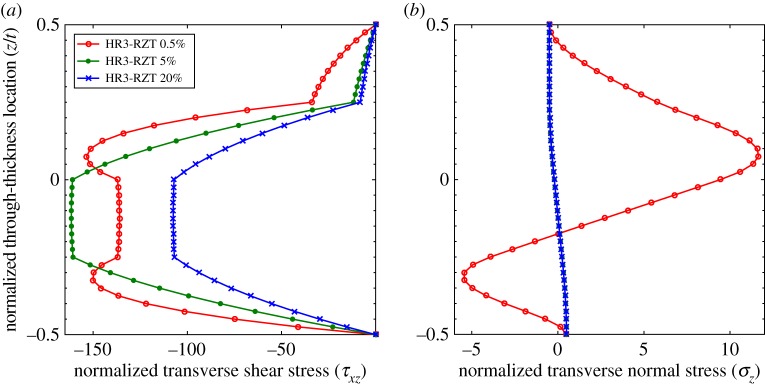
Laminate 1: comparison of through-thickness plots of normalized transverse shear stress ${\bar{\tau }}_{xz}$ and transverse normal stress ${\bar{\sigma }}_{z}$ at locations 0.5%, 5% and 20% of the plate span from clamped end *x*_*A*_ with *t*/*a*=1:200. (*a*) Transverse shear stress, ${\bar{\tau }}_{xz}$, and (*b*) transverse normal stress, ${\bar{\sigma }}_{z}$. (Online version in colour.)

**Figure 8.**
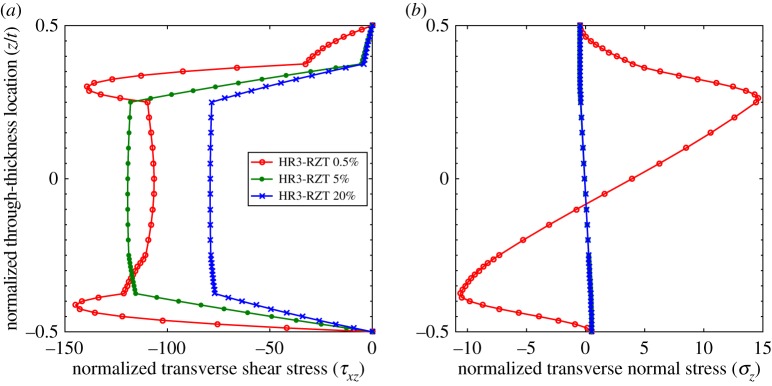
Laminate 2: comparison of through-thickness plots of normalized transverse shear stress ${\bar{\tau }}_{xz}$ and transverse normal stress ${\bar{\sigma }}_{z}$ at locations 0.5%, 5% and 20% of the plate span from clamped end *x*_*A*_ with *t*/*a*=1:200. (*a*) Transverse shear stress, ${\bar{\tau }}_{xz}$, and (*b*) transverse normal stress, ${\bar{\sigma }}_{z}$. (Online version in colour.)

The through-thickness plots of ${\bar{\tau }}_{xz}$ and ${\bar{\sigma }}_{z}$ at locations 0.5%, 5% and 20% for the thinner configurations (*t*/*a*=1:200) are compared in figures 7 and 8. Compared with the plots for the thicker laminates (*t*/*a*=1:10), the differences between the shape of the 5% and 20% curves for both ${\bar{\tau }}_{xz}$ and ${\bar{\sigma }}_{z}$ are negligible. Because the transverse shear force varies with spanwise location, the magnitudes of ${\bar{\tau }}_{xz}$ in figures 7*a* and 8*a* are different, but the overall through-thickness shape remains the same. A visible difference however exists between the curves of ${\bar{\tau }}_{xz}$ and ${\bar{\sigma }}_{z}$ at the 0.5% and 5% locations. This confirms the previous observations regarding figure 6*b* that the length of the boundary layer at the clamped edge decreases with the thickness-to-length ratio.

**Figure 9.**
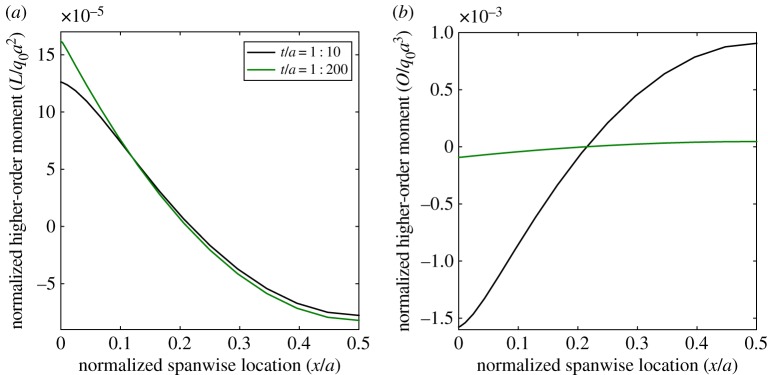
Axial variation of normalized ZZ moment *L*/*q*_0_*a*^2^ and higher-order moment *O*/*q*_0_*a*^3^ for laminate 1 with reduced transverse orthotropy ratio *G*_13_/*G*_23_= 1.01:1 of material p. Only half of the plot is shown due to the symmetry condition at *x*/*a*=0.5. (*a*) ZZ moment, *L*, and (*b*) higher-order moment, *O*. (Online version in colour.)

Second, consider the effects of maintaining the thickness-to-length ratio of laminate 1 at *t*/*a*=1:10 but reducing the ratio *G*_13_/*G*_23_ of material p from 2.5:1, as originally defined in table 2, to a lesser ratio of 1.01:1. The transverse shear modulus orthotropy between the 0° and 90° layers is almost removed entirely in this configuration, and as expected this reduces the magnitude of the ZZ moment (figure 9*a*). Furthermore, compared with the original case of greater transverse orthotropy in figure 6*b*, the local boundary layer of ZZ moment *L* close to the supports also reduces. Therefore, the boundary layer effect associated with the ZZ moment not only reduces for a lower thickness-to-length ratio *t*/*a* but also when the transverse orthotropy ratio decreases.

**Figure 10.**
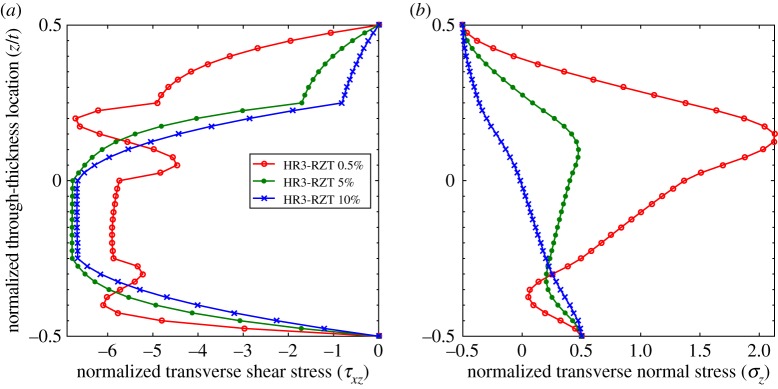
Laminate 1: comparison of through-thickness plots of normalized transverse shear stress ${\bar{\tau }}_{xz}$ and transverse normal stress ${\bar{\sigma }}_{z}$ at locations 0.5%, 5% and 10% of the plate span from clamped end *x*_*A*_ with *t*/*a*=1:10 and reduced transverse orthotropy ratio *G*_13_/*G*_23_=1.01:1 of material p. (*a*) Transverse shear stress, ${\bar{\tau }}_{xz}$, and (*b*) transverse normal stress, ${\bar{\sigma }}_{z}$. (Online version in colour.)

However, figure 10 shows that the through-thickness shapes of ${\bar{\tau }}_{xz}$ and ${\bar{\sigma }}_{z}$ do undergo changes in shape at different locations from the clamped boundary, even when the ZZ moment is benign. This particular boundary layer effect relates to the higher-order moment $O=\int z^{2}\sigma_{x}\;dz$ which becomes the dominant factor when ZZ effects are negligible. The axial distribution of the higher-order moment *O* in figure 9*b* shows that a small boundary layer occurs close to the supports that manifests itself by *O*_,*x*_≈0 at the boundary. This local change in slope modifies the *z*-wise stress profiles between locations 0.5% and 5% shown in figure 10.

**Figure 11.**
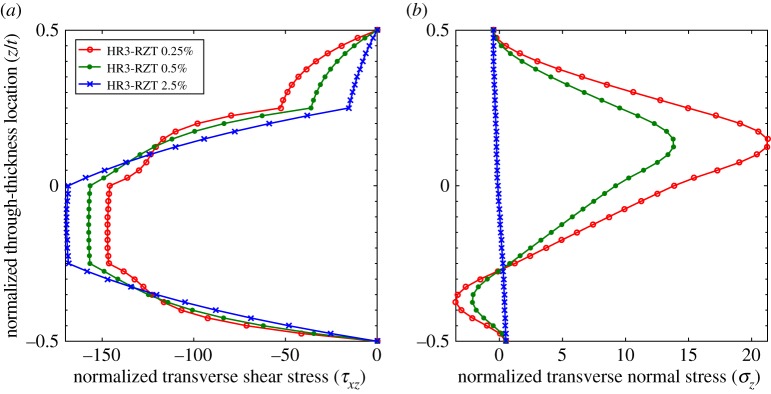
Laminate 1: comparison of through-thickness plots of normalized transverse shear stress, ${\bar{\tau }}_{xz}$ and transverse normal stress, ${\bar{\sigma }}_{z}$ at locations 0.25%, 0.5% and 2.5% of the plate span from clamped end *x*_*A*_ with *t*/*a*=1:200 and reduced transverse orthotropy ratio *G*_13_/*G*_23_=1.01:1 of material p. (*a*) Transverse shear stress, ${\bar{\tau }}_{xz}$, and (*b*) transverse normal stress, ${\bar{\sigma }}_{z}$. (Online version in colour.)

Finally, combining the reduced transverse orthotropy ratio with a lower thickness-to-length ratio (*t*/*a*=1:200) basically eliminates the local boundary layer in higher-order moment *O* (figure 9*b*). The through-thickness plots of the transverse stress fields ${\bar{\tau }}_{xz}$ and ${\bar{\sigma }}_{z}$ in figure 11 show that a benign boundary layer effect remains; the overall change in shape considerably reduces and manifests itself closer to the boundary, i.e. within *x*/*a*∈[0%,2.5%].

In conclusion, boundary layer effects at clamped edges can arise from higher-order membrane moments or from higher-order ZZ moments. These local effects scale in proportion to their relative magnitude of the respective moments on the global behaviour of the structure remote from any edges. When ZZ moments are important, such as for sandwich panels, the associated boundary layer effects dominate. The influence of ZZ effects generally increases towards clamped edges and these higher-order moments are therefore important even for non-sandwich constructions. For laminated structures where the ZZ effects are negligible throughout, such as composite laminates with thin plies evenly distributed through the thickness, or laminae with negligible transverse shear modulus orthotropy, boundary layer effects associated with higher-order membrane moments play a more important role.

These boundary layers can play a significant role in interlaminar failure initiation such as delamination. A popular metric for predicting the onset of delamination in layered composites is the quadratic failure criterion of Camanho *et al.* [40]

3.2 $$f={\left(\frac{\left\langle \sigma_{z}\right\rangle }{N}\right)}^{2}+{\left(\frac{\tau_{xz}}{S}\right)}^{2}+{\left(\frac{\tau_{yz}}{T}\right)}^{2},$$

where *N* is the interlaminar tensile strength, and *S* and *T* are the interlaminar shear strengths. Delamination initiation is assumed to occur when *f*≥1. Macaulay brackets 〈〉^1^ are used because compressive transverse normal stresses do not contribute to the initiation of delaminations. In the problem considered here, *τ*_*yz*_=0 such that delamination initiation is driven by *σ*_*z*_ and *τ*_*xz*_ at the interface between two plies with different material properties. Even when we consider the configuration of laminate 1 with the most benign boundary effect (*t*/*a*=1:200, *G*_13_/*G*_23_=1.01:1 in figure 11), the maximum transverse normal stress *σ*_*z*_ at the interface between the top 0° and adjacent 90° layer (*z*/*t*=0.1667) is increased by two orders of magnitude between 0.25% and 2.5% from the edge. As a result, the boundary layer plays a crucial role in the failure initiation event and needs to be appropriately considered in analysis and design.

In fact, within the framework of RZT resin-rich regions between layers can be modelled explicitly by inserting a thin isotropic interlayer in the stacking sequence. A continuum damage law can then be chosen to govern the initiation of damage and the degradation process within resin-rich regions, and thereby model the sliding of the resulting sublaminates across each other via ZZ kinematics [41,42].

### (b) Localized stress gradients driven by tow steering

Previous studies [29,43] show that non-intuitive transverse shear stress profiles occur for straight-fibre laminates at the corner of intersecting clamped edges. The results in this section show that stiffness variations in the plane of a plate can induce the same boundary layer effects but remote from any edges. Most importantly, these non-intuitive stress gradients may adversely affect the damage tolerance of tow-steered laminates.

To study the effect in straight-fibre laminates, consider a square [0/90/0] laminate with in-plane dimensions *a*=*b* and thickness to characteristic length ratio of *t*/*a*=1:10, clamped along all four edges and loaded by a uniform pressure on the top surface. The bending behaviour of this laminate is readily investigated using the HR plate model published recently by the present authors [36]. The dual clamped condition at each corner of the plate induces a particular boundary layer with non-intuitive transverse shear stresses, and this particular behaviour does not occur in cylindrical bending of plates.

**Figure 12.**
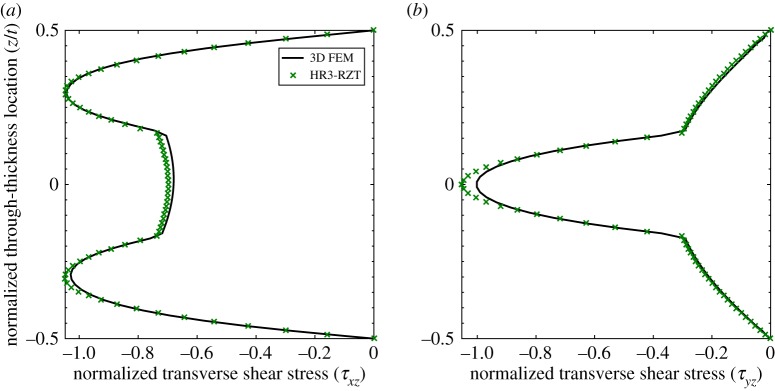
Through-thickness distribution of the normalized transverse shear stresses *τ*_*xz*_ and *τ*_*yz*_ at location (0.066*a*,0.066*b*,*z*) of a [0/90/0] laminate. The two plots are not identical, even though the loading condition produces a doubly symmetric displacement field, because of the orthotropy of the constituent materials. (*a*) Transverse shear stress, *τ*_*xz*_, and (*b*) transverse shear stress, *τ*_*yz*_. (Online version in colour.)

Figure 12 shows the through-thickness plots of the transverse shear stresses at a distance of (*x*,*y*)=(0.066*a*,0.066*b*) from one of the corners of the plate. The stress distributions of the HR3-RZT and 3D FEM model closely match at this location. Note, the transverse shear stresses *τ*_*xz*_ and *τ*_*yz*_ are not identical at this point, even though the loading condition produces a doubly symmetric displacement field, because of the orthotropy of the constituent layers. Hence, in the *xz*-plane the middle 90° layer is more compliant and the stresses redistribute towards the outer 0° layers via the ZZ effect (figure 12*a*). In the *yz*-plane, the situation reverses and the stress distributes to the stiffer central layer (figure 12*b*).

**Figure 13.**
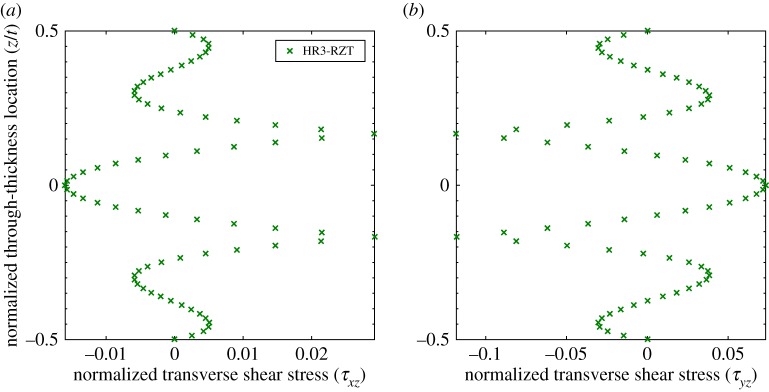
Through-thickness distribution of the normalized transverse shear stresses *τ*_*xz*_ and *τ*_*yz*_ at location (0,0,*z*) of a [0/90/0] laminate. Note the change of sign of the transverse shear stresses through the thickness. (*a*) Transverse shear stress, *τ*_*xz*_, and (*b*) transverse shear stress, *τ*_*yz*_. (Online version in colour.)

Conversely, figure 13 shows the through-thickness plots of the transverse shear stresses at a corner of the plate, i.e. at (*x*,*y*)=(0,0). It is apparent that the transverse shear plots in the corner differ considerably from the plots adjacent to the corner. Adjacent to the corner in figure 12, we observe the classical result of single sign, piecewise parabolic transverse shear stresses, i.e. the applied pressure loading on the top surface causes the cross-section to shear in one direction only. However, at the corner in figure 13, the model solution shows that both transverse shear stresses change sign through the thickness, i.e. some parts of the cross-section shear in one direction, whereas other parts shear in the opposite direction. This non-intuitive stress distribution arises from the strong dual boundary condition of two coincident clamped edges at the corner point. Small movements away from the corner, as shown in figure 12, completely eliminate this phenomenon suggesting the presence of a boundary layer effect.

Similar plots are shown in the works by Vel & Batra [43] and Shah & Batra [29] but these authors did not point out or study the peculiarity of these stress fields in further detail. As is shown below, the same effects can be replicated in variable-stiffness plates at locations considerably removed from any boundaries. Thus, boundary layers that occur in the vicinity of dual boundary conditions for straight-fibre laminates can be also induced by varying the material properties alone.

For example, consider a multilayered plate with thickness to characteristic length ratio *t*/*a*=1:20, comprising purely variable-stiffness composite layers, pinned (free to rotate) at both ends *x*_*A*_=0 and *x*_*B*_=*a*=250 mm, and subjected to a uniformly pressure equally divided between the top and bottom surfaces ${\hat{P}}_{b}=-{\hat{P}}_{t}=50\;\mathrm{kPa}$.

Table 3 summarizes two balanced and symmetric, variable-stiffness lay-ups VS X and VS Y that are analysed using the HR3-RZT model. The two laminates feature eight tow-steered plies of equal thickness manufactured using the IM7 8552 carbon-fibre reinforced plastic defined in table 2.

**Table 3.** Stacking sequences and material properties of two tow-steered laminates used to investigate localized stress fields due to in-plane stiffness variations. The notation of the variable-stiffness lay-ups follows ref. [38], eq. (6.1), p. 119 and the subscript ‘s’ signifies symmetry of the lay-up about the midplane.

laminate	lay-up	*t*_*ply*_ (mm)
VS X	[〈90|0〉/〈−90|0〉/〈45|−45〉/〈−45|45〉]_*s*_	1.5625
VS Y	[〈90|20〉/〈45|−25〉/〈−90|−20〉/〈−45|25〉]_*s*_	1.5625

A 3D FEM model in Abaqus was also implemented that featured the 250 mm long (*x*-direction), 1000 mm wide (*y*-direction) and 12.5 mm thick (*z*-direction) plate that was meshed using a total of 95 880 C3D8R elements with 799 elements in the *x*-direction, 120 elements in the *z*-direction, i.e. 15 elements per ply. The plane strain condition in the *y*-direction was enforced by the use of a single element in the *y*-direction and boundary conditions preventing the lateral sides from expanding.

**Figure 14.**
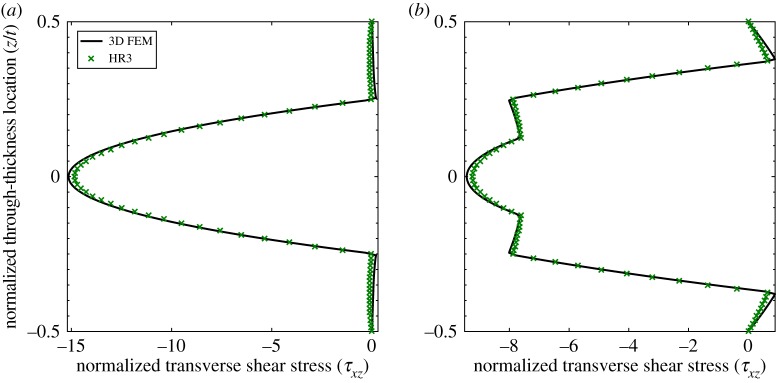
Through-thickness distribution of the normalized transverse shear stress *τ*_*xz*_ (at *x*=*a*/4). Note the change of sign of the transverse shear stress through the thickness. (*a*) Laminate VS X and (*b*) laminate VS Y. (Online version in colour.)

Figure 14 plots the through-thickness profile of the normalized (see equation (3.1)) transverse shear stress ${\bar{\tau }}_{xz}$ for laminates VS X and VS Y at the quarterspan (*x*=*a*/4) of the plate. The plots show that the 3D FEM solution from Abaqus and the HR3-RZT results correlate closely throughout the entire thickness.

For both laminates, the external surface layers shear in one direction, whereas the internal layers shear in the opposite direction. Hence, this behaviour qualitatively compares to the results shown in figure 13. In this latter case, the two coincident clamped edges induced strong localized boundary layers in the transverse shear stress profiles. However, for the two tow-steered plates VS X and VS Y, the transverse shearing reversal occurs at the quarterspan, i.e. significantly removed from any localized boundary condition.

The physical reason of why this occurs in tow-steered plates is readily explained by investigating the transverse shear stress equation of the HR model with ${\hat{T}}_{b}=0$:

3.3 $$\tau_{xz}^{\left(k\right)}=\frac{\partial }{\partial x}[\{-{\bar{Q}}^{\left(k\right)}{\text{g}}^{\left(k\right)}+\alpha^{\left(k\right)}\}\text{s}F].$$

In equation (3.3), the only quantities that influence the layerwise sign of the transverse shear stress are ply stiffness ${\bar{Q}}^{\left(k\right)}$ and integration constant ***α***^(*k*)^, where the latter is a function of the ply stiffness terms ${\bar{Q}}^{\left(k\right)}$ itself. All other terms, ***s*** and F, are equivalent single-layer quantities that are the same for all plies. For any material that satisfies the second law of thermodynamics the definition ${\bar{Q}}^{\left(k\right)}>0$ holds, but for tow-steered laminates the derivative $\partial {\bar{Q}}^{\left(k\right)}/\partial x\neq 0$ and can therefore be positive for some layers and negative for others. Hence, the sign of the transverse shear stress can change based on the local rate of change of the material stiffness ${\bar{Q}}^{\left(k\right)}$.

The practicality of these non-intuitive transverse shear stress profiles is yet to be determined. Perhaps, these localized stress fields can be used for actuation purposes in morphing structures or for sensing of interlaminar damage sites. On the contrary, the presence of such stress fields could be detrimental to the damage tolerance of tow-steered laminates. The external loading and boundary conditions fully define the transverse shear force, i.e. the through-thickness integral of the transverse shear stress, and hence the shear force is independent of the stacking sequence or material properties of the laminate. If the overall transverse shear force is to remain constant, locally positive transverse shear stresses must result in increased negative transverse shear stresses at other locations of the cross-section and vice versa. As the magnitude of transverse shear and normal stresses drive the debonding of layers in laminated composites, these locally accentuated levels of transverse stresses could initiate premature delamination failure.

## 4. Conclusion

This work demonstrates that the proposed higher-order model derived from the Hellinger–Reissner mixed variational principle accurately predicts local variations in the 3D stresses towards clamped edges. In this model, the equilibrium of stresses and all surface traction conditions are enforced explicitly. This feature combined with a strong-form solution technique and a spectral collocation grid allows accurate recovery of the steep stress gradients towards clamped edge.

The observed boundary layer effects arise due to local variations in the higher-order stress resultants. In fact, the relative significance of higher-order distortions increases towards clamped boundaries such that a higher-order model is critical in capturing the observed phenomena. For sandwich plates the boundary layer effects are driven by local variations in the ZZ moment. For composite laminates variations in the higher-order membrane moment dominate the boundary layer. Hence, the relative significance of the boundary layer is a function of both layerwise differences in transverse shear moduli and the thickness-to-length ratio of the laminate.

The boundary layers towards clamped edges accentuate the transverse shear and transverse normal stresses through the thickness of the laminate. As these through-thickness stresses play a critical role in delamination initiation, robust modelling techniques that account for deleterious stress fields induced by boundaries are essential.

In fact, the deleterious stress fields are not only restricted to the edges of laminates comprising straight-fibre reinforced plastics. This work shows that non-intuitive localized stress fields can also occur in tow-steered laminates remote from any boundaries. The significance of this finding is twofold

(i) Optimization studies on tow-steered laminates have predominantly focused on improving the structural stability of plates [44–46], shells [47] and stiffened panels [48]. The fibre paths are tailored to redistribute in-plane stresses over the planform to preempt the onset of buckling. The present findings suggest that *in-plane* stiffness variations facilitated by tow-steering can be used to tailor the *through-thickness* stresses. Pertinent questions worth answering are to what extent the transverse stresses can be influenced via stiffness variations? Could the through-thickness stresses be tailored to reduce the likelihood of delaminations in critical areas? Perhaps, the reversal in transverse shear stress through the plate thickness could be used for passive actuation of morphing structures by means of relieving residual stresses?
(ii) Current design studies on the buckling and post-buckling optimization of tow-steered laminates rarely account for transverse shear stresses because design codes deem these effects to be negligible for thin-walled structures. However, if non-intuitive through-thickness stresses, such as the transverse shear stress reversals described in this study, are deleterious to the damage tolerance of tow-steered laminates, and these effects occur remote from boundaries or other geometric discontinuities, then the effects need to be accounted for by changes in the design guidelines. In this case, higher-order modelling of non-classical effects is required throughout the entire structure, not just in areas with local boundary features, and computationally efficient 2D modelling techniques will become critical for safe design of these laminates.

## Footnotes

1 〈*x*〉=0 for *x*≤0 and 〈*x*〉=*x* for *x*>0.

## Data accessibility

All Matlab programs and plots are available through the University of Bristol data repository http://data.bris.ac.uk/data/ or via the doi:10.5523/bris.1rpezb367id5212bkarcz5a8iz.
